# Review of the prevalence of postnatal depression across cultures

**DOI:** 10.3934/publichealth.2018.3.260

**Published:** 2018-07-20

**Authors:** Siti Roshaidai Mohd Arifin, Helen Cheyne, Margaret Maxwell

**Affiliations:** 1 Department of Special Care Nursing, International Islamic University Malaysia, Kuantan, Pahang, Malaysia; 2 Nursing, Midwifery and Allied Health Professional (NMAHP) Research Unit, University of Stirling Scotland, United Kingdom

**Keywords:** prevalence, postnatal women, postnatal depression, cultures

## Abstract

The purpose of this review was to examine articles related to recent epidemiological evidence of the prevalence of maternal postnatal depression (PND) across different countries and cultures and to identify specific epidemiological studies that have been carried out exclusively in Malaysia on the prevalence of maternal PND. The review was undertaken in two stages, an initial review and an updated review. At both stages systematic literature searches of online databases were performed to identify articles on the prevalence of maternal PND. A total of 124 articles concerning research conducted in more than 50 countries were included in the final analysis. There were wide variations in the screening instruments and diagnostic tools used although the Edinburgh Postnatal Depression Scale (EPDS) was the most common instrument applied to identify PND. The prevalence of maternal PND ranged from 4.0% to 63.9%, with Japan and America recording the lowest and highest rates, respectively. Within continents, a wide variation in reported prevalence was also found. The reported rates of maternal PND in Malaysia were much higher than that previously documented with a range of 6.8–27.3%. This review indicated that the widely cited prevalence of maternal PND of 10–15% underestimates rates of PND worldwide. The reasons for this variability may not be fully explained by review methods. Future studies should evaluate the nature of women's PND experiences across cultures to explain these wide variations.

## 1. Introduction

Postnatal depression (PND) is one of the most common causes of maternal distress representing a considerable public health problem affecting the mother, her baby, and her family [1]. Within the postnatal period, there is an increase in the physical and emotional demands on the woman and the debility associated with PND may impinge on her capacity as a mother for example, to care for and bond with her new-born. In some instances the woman may be less engaged, and may even react negatively towards the child [2]. Without diagnosis and treatment, maternal PND may affect her ability to participate in normal activities and interfere with her family and other social relationships. These problems can compromise maternal-infant relationships which may be associated with poor child cognitive and behavioral and social development [3]–[6]. Partners of women with PND have also been shown to be at risk of poor mental health [7]–[9].

Although these experiences are commonly shared by women across cultures, experiences of PND are not fully shared or similarly expressed by women across the world, with some experiences being more common in certain cultures or countries. For instance, whilst social circumstance and biophysical stressors were described as factors contributing to PND in many countries, issues of culture and traditions were more common in Asian women [10],[11]. Considering ways of reducing their distress, Asian women were more likely to describe religious beliefs and social support, whereas European women talked about recognizing their own needs and personal adjustment such as keeping busy and getting out the house every day [10]–[17]. These findings suggest that culture can affect the way the women interpret their experience of PND, the causes of PND and influences on their coping strategies. Culture can play an important role in women's experience of pregnancy and after childbirth as it is comprised of several shared ideas, values, perspectives, beliefs, and “perceived standards” for emotional and behavioral responses [18].

The prevalence of PND is highly variable in non-western countries and its manifestations may vary across cultures [19],[20]. For instance, previous reviews have shown that the prevalence of PND ranged widely from 0 to 60% globally, and from 3.5 to 63.3% in Asian countries [20],[21]. These findings suggest that there is a link between the conceptualization of PND and culture yet there is a lack of recent evidence on PND across different countries. Taking an example of Malaysia as an example of a non-western country the aims of this review are twofold:

- To provide recent epidemiological evidence of the prevalence of maternal PND across different countries and cultures.
- To identify specific epidemiological studies that have been carried out exclusively in Malaysia on the prevalence of maternal PND.

## 2. Methods

The review was undertaken in two stages: The initial review and a more recent updated review. The initial review was conducted based on the search terms used by Halbreich and Karkun [21]. The updated review was conducted with an improved search strategy. Both reviews were conducted by the first author (MA). Articles were selected based on the inclusion and exclusion criteria (Table 1). Discussions with the second and third author were conducted to resolve any discrepancies in decisions about excluding or including articles.

**Table 1.** Initial and updated review of prevalence of postnatal depression.

	Initial reviews (2006–2014)	Updated reviews (2010–2016)
Databases	CINAHL, MEDLINE, PubMed, PsycArticles, PsycINFO, Web of Science, and The Cochrane Library.	CINAHL, MEDLINE, PubMed, PsycINFO and ASSIA.
Keywords	Prevalence, postnatal depression, and postpartum depression.	Incidence, prevalence, postnatal depression, postpartum depression, depression, maternal mental health, depressive disorders, puerperal disorders, emotional distress, low mood disorders, after childbirth, psychological distress.
Inclusion criteria	Peer reviewed articles published from 2006–2014, studies that report prevalence of PND within 1–12 months following childbirth, full text available, and English/Malay language publications.	Peer reviewed articles published from 2010–2016, studies that report prevalence of PND within 1–12 months following childbirth, and English/Malay language publications.
Exclusion criteria	Review papers, and PND and/or postnatal women were not the focus of the study.	Review papers, PND and/or postnatal women were not the focus of the study, studies within psychiatric populations, and studies that were conducted among high risk groups of women.
Total studies included in the final analysis	39	104

### 2.1. Search strategies

Electronic databases used in both two reviews were: CINAHL, MEDLINE, PubMed, and PsycINFO. In the updated review, PsycArticles were not used as articles in this database are also available in PsycINFO, whereas Web of Science and The Cochrane Library were not used as articles in both databases are also available in ASSIA.

**Figure 1.**
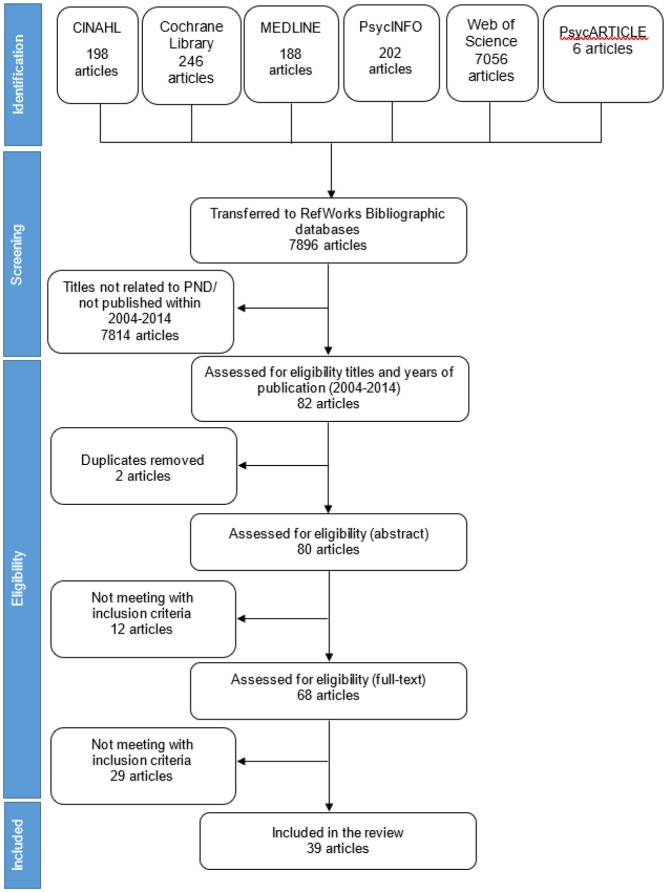
Flow diagram of the search strategy for prevalence of postnatal depression (initial review).

The search strategy for both reviews can be seen in Figure 1. Searches were conducted using the following keywords: Incidence, prevalence, postnatal depression, postpartum depression, depression, maternal mental health, depressive disorders, puerperal disorders, emotional distress, low mood disorders, after childbirth, psychological distress. Keywords were also combined using AND and OR to identify as many articles as possible on the prevalence of maternal PND.

### 2.2. Inclusion and exclusion criteria

In both reviews, PND is defined as “any depressive symptomatology occurring within the first postnatal year”. However, to avoid inclusion of postnatal blues in the reported prevalence, the time frame used in these reviews was 1–12 months following childbirth. For studies that assessed the prevalence at more than one-time point only prevalence within 1–12 following childbirth was included in this review. For studies that included more than one time point within 1–12 months all reported prevalence was included in the review. Studies undertaken within the year 2006–2016 were included because they were more likely to reflect the current state of knowledge on PND. The largest review involving worldwide studies by Halbreich and Karkun [21] only included articles up to 2005. High risk populations (some population groups are at considerably higher risk of developing PND than others such as women with unsuccessful attempted abortions or women who gave birth prior to an earthquake) and psychiatric population (women with known psychiatric diagnosis such as schizophrenia or anxiety disorders) were excluded because their risks developing PND may reach 40–60% [22]. This present review was conducted using scoping review methodology reported in Joanna Briggs Institute Reviewers' Manual 2015 Methodology for JBI Scoping Reviews [23].

## 3. Results

**Figure 2.**
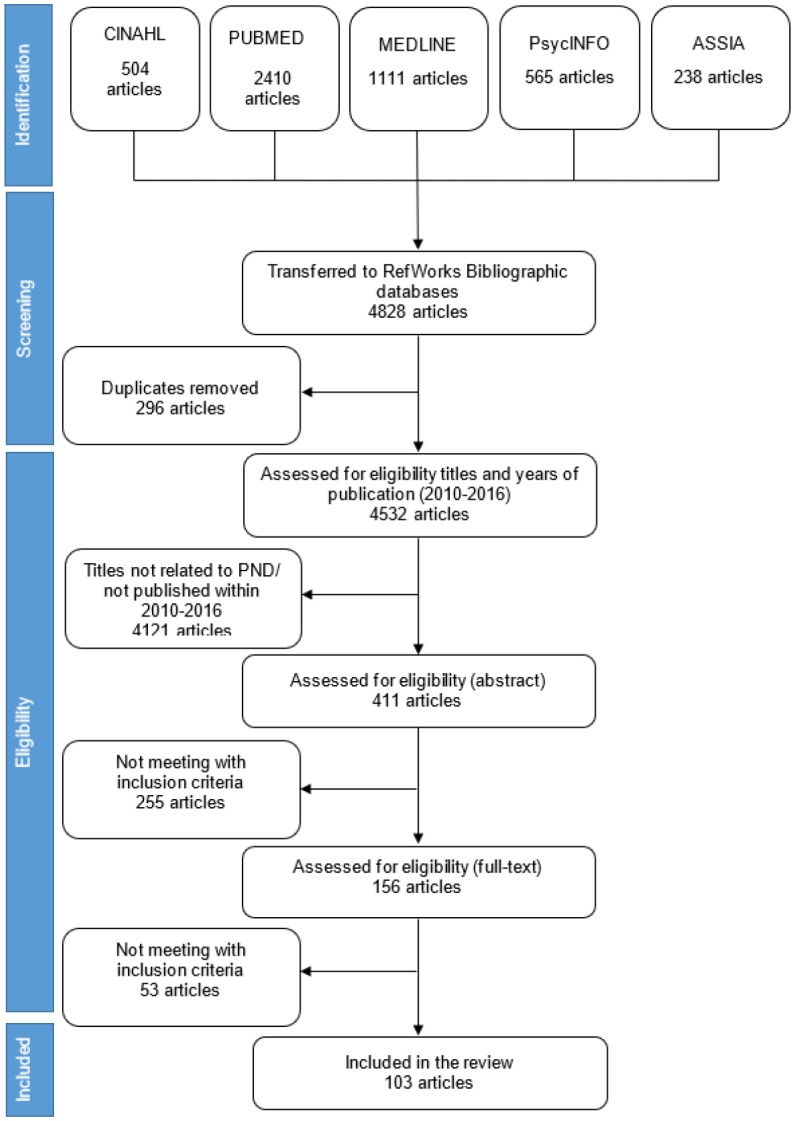
Flow diagram of search strategy for prevalence of postnatal depression (updated review).

Findings presented in this section are based on the summary of both the initial and updated reviews. The initial review identified 7896 articles, screened 80 abstracts, and identified 68 full text papers for inclusion. The updated review identified 4828 articles, screened 411 abstracts, and identified 156 full text papers for inclusion. Figures 1 and 2 show the flow diagrams of search strategies used in the initial and updated reviews, respectively.

The most common reasons for excluding articles in both reviews were that they; did not relate to PND/did not publish within 2006–2014 (initial review) or 2010–2016 (updated review), were not conducted within 1–12 months following childbirth, were not focused on maternal PND, did not report prevalence of maternal PND, were review papers, were conducted among high risk groups of women, were not in English/Malay language, were duplicate studies, were within a psychiatric population, and were conducted among fathers with PND.

Whilst the initial review finally included 39 articles, 102 articles were included in the updated review. A total of 17 studies were duplicates (found in both reviews) and ultimately 124 articles were included in the final analysis. These studies were conducted in more than 50 countries. Described in relation to continents, 58 studies were conducted in Asia, 22 in North America and seven in South America, 23 in Europe, nine in Australia and New Zealand, and five in Africa. Data extracted from each included study were screening instruments, sample size, time of assessment, study design, study setting, and prevalence of maternal PND (Table 2).

There were wide variations in the screening instruments and diagnostic tools used, although the Edinburgh Postnatal Depression Scale (EPDS) was the most common instrument applied to identify maternal PND. All studies used self-report measure (e.g. EPDS, Patient Health Questionnaire (PHQ), Center for Epidemiological Studies Depression (CES-D) except 11 studies that used diagnostic interviews (e.g. Mini-International Neuropsychiatric Interview (MINI)) or International Statistical Classification of Diseases and Related Health Problems 10th Revision (ICD-10)). A total of 87 studies used translated versions (75 studies used the available translated and validated version, 11 studies used the version that was translated and validated by the authors themselves, and one used non-validated version), 34 studies used the original English version, and three studies did not clearly mention whether the measure was translated. Most studies included were cohort or cross sectional, however, study sample sizes and the timing of assessments varied (39-410 367 participants and 30 days to one year after giving birth) across the 124 studies. The total numbers of participants by continent are: Asia (28,718), North America (309,296), South America (5,934), Europe (426,219), Australia and New Zealand (13,737), and Africa (1,511). The majority of studies were conducted in community settings/outpatient maternity clinics, although there were also studies which analyzed data from large population-based surveys.

Overall, the prevalence of PND ranged from 4.0% to 63.9%, with Japan and America recording the lowest and highest rates of PND, respectively [9],[41]. Within continents, a wide variation in reported prevalence was also found. The prevalence of PND varied from 4.0–48.3% in Asia [117],[137], 5.0–63.9% in America [38],[41], 4.4–22.8% in Europe [78],[61], 7.2–50.3% in Africa [54],[57], 6.0–32.8% in Australia [86],[87], and 7.6–30.9% in New Zealand [90]. Wide variations in the rates of PND have also been reported within Asian countries. For instance, the reported prevalence of PND ranged from 15.8–46.9% in India [101],[103] and 9.4–27.4% in China [95],[96].

In Malaysia, the prevalence of PND ranged from 6.8–27.3% [122],[123]. A total of five Malaysian studies were published between 2006 and 2016 and were included in this review. The prevalence reported in these studies differed as there were differences in study instruments, sample size, time of assessment, study design, and study setting. The EPDS with a cut-off point of 12 was used in all studies except one [122]. The times of assessments used to assess PND in these studies were 4–6 months following childbirth. The sample sizes ranged from 293–979 with a total of 2458 postnatal women involved in the five studies. Three of the studies used a cross sectional design; one was a prospective cohort and one was a population survey. Three of these Malaysian studies were conducted in maternal and child health (MCH) clinics, whereas two of them were conducted in postnatal clinics at a teaching hospital. Whilst three of these studies focussed mainly on Malay women, two included women from other cultural backgrounds, such as Chinese, Indian, and other ethnic minorities.

**Table 2.** Studies on prevalence of postnatal depression.

Region/Country	Authors	Instruments	Sample size (postnatal women)	Time of assessment (after delivery)	Study design	Study setting	Prevalence (%)
America							
US	Levine et al. [24]	ICD-9-CM (English)	161,454	8 weeks, 1 year	Retrospective cohort	Information from the Registry	16.2
	Stone et al. [25]	PHQ-2 (English)	5,395	2, 6 months	Surveys (secondary analysis)	Massachusetts Pregnancy Risk Assessment Monitoring System (MA-PRAMS) 2007–2010 data	14.9
	Lynch and Prasad [26]	PRAMS questionnaire (English)	40,337	2–4 months	Cross-sectional (secondary analysis)	population-based national data from PRAMS	13.3
	Sidebottom et al. [27]	PHQ-9 ≥ 10 (English)	594	≥ 4 weeks	Prospective	Community health centres	6
	Abbasi et al. [28]	EPDS ≥ 12 (English)	2972	1 month	Prospective cohort	Hospitals, obstetricians' offices and clinics, and targeted mailings	5.1
	Dolbier et al. [29]	EPDS ≥ 10 (English)	299	1, 6 months	Cohort	Community child health network	17.5, 17.4
	Pooler et al. [30]	PHQ-2 (English)	75,234	2–6 months	Surveys (secondary analysis)	Pregnancy Risk Assessment Monitoring System (PRAMS) data	13.8
	Schachman and Lindsey [31]	PDSS ≥ 14 (English)	71	8 weeks	Comparative descriptive	Military Immunization Clinic	50.7
	Sweeney et al. [32]	EPDS ≥ 12 (English)	46	2 months	Prospective cohort	Obstetrics and gynaecology offices and midwifery offices	10.9
	Wisner et al. [33]	EPDS ≥ 10 (English)	10,000	4–6 weeks	Sequential case series	obstetric hospital	14.0
	Dagher et al. [34]	EPDS (cut off point not mentioned) (English)	526	8 weeks	Prospective cohort	Home interview	6.5
	Gress-Smith et al. [35]	CES-D ≥ 24 (English)	132	5, 9 months	Longitudinal	Hospital	33, 38
	Kornfeld et al. [36]	US Preventive Services Task Force (English)	39	2 months	Retrospective (secondary analysis)	Academic-based paediatric primary care clinic (new mothers survey data)	15
			38	4 months			29
			31	6 months			26
	Beck et al. [37]	PDSS-Short Form ≥ 14 PHQ-2 (English)	1,566	1–12 months	National survey	Hospital	63
	Gjerdingen et al. [38]	PHQ-9 ≥ 10 (English)	464	2 months	Prospective cohort	Family medicine residency clinics and private paediatric clinics	7.1
			459	4 months			7.0
			455	6 months			5.0
			472	9 months			10.2
	***Wang et al. [39]***	***CES-D ≥ 16 (English)***	***1,364 families***	***1***	***Longitudinal***	***Hospitals***	***25.6***
				***6 months***			***16.3***
	Murphy et al. [40]	EPDS ≥ 10 (Translated, validated)	97	4–6 weeks	Cohort	Postnatal clinic	12
	Le et al. [41]	PDSS ≥ 60 (Translated, validated by the researcher)	220	6–8 weeks	Longitudinal	Clinics	63.9
	Sorenson et al. [42]	BDI-II (English)	71	6–7 months	An exploratory investigation	Daily newspaper listing parent(s) name and city of residence	15.7
	*Mcgrath et al. [43]*	*EPDS* ≥ 13 *(English)*	*139*	*2, 6 months*	*Longitudinal design*	Care provider's offices	*11, 15*
Canada	Verreault et al. [44]	EPDS ≥ 10 (English)	226	3 months	Cohort	Health centre and ultrasound department	16.4
	Dennis et al. [45]	EPDS ≥ 13 (English)	6,421	12 weeks	Cross-sectional survey	Data from the Maternity Experiences Survey of the Canadian Perinatal Surveillance System	8
Greenland	***Motzfeldt et al. [46]***	***EPDS ≥ 13 (Translated, validated by the researcher)***	***174***	***3 months***	***Cross-sectional***	***Primary health care***	***8.6***
Argentina	Mathisen et al. [47]	EPDS ≥ 10 (Translated, validated)	86	4–12 weeks	Cross sectional	Private health care centre	37.2
Brazil	Rebelo et al. [48]	EPDS ≥ 11 (Translated, validated)	177	30–45 days	Prospective cohort	Antenatal care unit	16.9
	Matijasevich et al. [49]	EPDS ≥ 11 (Translated, validated)	3,332	3, 12 months	Cohort	Maternity hospitals	34.8, 40.9
	***Melo et al. [50]***	***EPDS ≥ 12 (English)***	***555***	***4–6 weeks***	***Cross-sectional***	***Prenatal clinic of two public reference centres***	***10.8***
	***Lobato et al. [51]***	***EPDS ≥ 12 (Translated, validated)***	***811***	***46–75 days***	***Cross-sectional***	***Primary health care units***	***21.8***
				***76–105 days***			***37.5***
				***106–135 days***			***24.5***
	Pinheiro et al. [52]	EPDS ≥ 10 (Translated, validated)	397	9–12 weeks, 12 months	Cohort	Brazilian National System of Public Health	22.7
			366				24.6
Mexico	Lara et al. [53]	Structured Clinical Interview (SCID-I; PHQ ≥ 10 (Translated, validated by the researchers)	210	6 weeks, 6 months	Longitudinal	Hospital and community centre	11.4, 9.0
Africa							
Ghana and Ivory	Guo et al. [54]	PHQ-9 ≥ 10 (Translated, validated)	654	3 and 12 months	Cohort	Hospital	11.8, 16.1 and 8.9, 7.2
*Morocco*	*Alami et al. [55]*	*EPDS ≥ 13 M.I.N.I.* (Translated, validated)	*100*	*From the first trimester of pregnancy to 9 months after delivery*	*Prospective cohort study*	Primary healthcare setting	17
*Nigeria*	*Abiodun [56]*	*EPDS ≥ 9* (Translated, validated by the researcher)	*360*	*6 weeks postnatal*	*Cross sectional*	Primary health care	*18.6*
South Africa	Stellenberg and Abrahams [57]	EPDS (cut off point not mentioned) (English)	159	6, 10 or 14 weeks	Cross-sectional	Primary health care clinics	50.3
Sudan	Khalifa et al. [58]	EPDS ≥ 12 (Translated, validated by the researcher)	238	3 months	Cross-sectional	Antenatal clinic public tertiary hospitals	9.2
Europe							
*England*	***Leahy-Warren et al. [59]***	***EPDS ≥ 12 (English)***	***410,367***	***6, 12 weeks***	***Longitudinal***	***Community sample***	***13.2, 9.8***
France	Gaillard et al. [60]	EPDS ≥ 12 (Translated, validated)	264	6, 8 weeks	Prospective	Public maternity unit	16.7
Greece	Lambrinoudaki et al. [61]	EPDS ≥ 11 (Translated, validated)	57	6 weeks	Cross-sectional	University hospital	22.81
	Koutra et al. [62]	EPDS ≥ 13 (Translated, validated)	438	8 weeks	Prospective cohort	Maternity clinics	13
	*Leonardou et al. [63]*	*GHQ, BDI and WHOQOL scores* (Translated, validated)	*81*	*2 months*	*Prospective cohort study*	*Maternity hospitals*	*12.4*
Germany	Goecke et al. [64]	EPDS ≥ 9 mild EPDS ≥ 12 medium to severe (Translated, validated)	159	6 months	Prospective	Obstetrics and gynaecology clinic	10.1, 1.9
	*Zaers et al. [65]*	*EPDS ≥ 13* (Translated, validated)	*47*	*6 weeks, 6 months*	*Prospective longitudinal study*	*Hospital*	*22, 21.3*
Hungary	Kozinszky et al. [66]	Leverton questionnaire (LQ) score of ≥ 12 (Translated, validated)	Year 1996: 2,333 Year 2006: 1,619	6–10 weeks	Longitudinal	Pregnancy-care centres	15.0 17.4
Italy	Elisei et al. [67]	EPDS ≥ 13 (Not mentioned)	85	3 months	Cohort	Obstetrics and gynaecology clinic	16.7
	***Giardinelli et al. [68]***	***EPDS ≥ 10 (Translated, validated)***	***590***	***3 months***	***Prospective cohort***	***Obstetrics and gynaecology clinic***	***13.2***
	***Banti et al. [69]***	***EPDS ≥ 13 SCI DSM-IV (Translated, validated)***	***1,066***	***1, 3, 6, 9, 12 months***	***Longitudinal***	***Hospital***	***9.6***
Netherlands	Meijer et al. [70]	EPDS ≥ 10 (Translated, validated)	1,276	4–7 months	Prospective cohort	Obstetric care	8.5
	*Meltzer-Brody et al. [71]*	*EPDS ≥ 12 (Not mentioned)*	*682*	*4–12, 12 weeks*	*Large cohort study*	*Subjects were from the Netherlands Study of Depression and Anxiety (NESDA)*	*13, 10*
*Norway*	*Glavin et al. [72]*	*EPDS ≥ 10* (Translated, validated)	*2,227*	*6 weeks after delivery*	*Cross-sectional study*	*Well baby clinics*	*10.1*
Portugal	Figueiredo and Conde [73]	EPDS ≥ 10 (Translated, validated)	260 couples (260 women)	10–14 weeks	Cohort	Obstetrics and gynaecology clinic	11.1
	***Maia et al. [74]***	***BDI-II ≥ 11, PDSS ≥ 63***	***386***	***3-months***	***Longitudinal***	***Local health medical centres***	***13.0, 16.8***
	Marques et al. [75]	Diagnostic Interview for Genetic Studies (DIGS), BDI-II (Translated, validated)	382	3 months	Cross-sectional	Mother's local medical centre or homes	11.5, 16.6
Poland	Dudek et al. [76]	EPDS ≥ 12 (Translated, non-validated)	344	6, 12 weeks	Cross-sectional	Obstetrics and gynaecology clinic	16
Serbia	Dmitrovic et al. [77]	EPDS ≥ 12 Hamilton Depression Rating Scale (English)	195	6–8 weeks	Cross-sectional	Obstetrics and gynaecology clinic	11
Spain	Escriba-Aguir and Artazcoz [78]	EPDS ≥ 11 (Translated, validated)	420	3, 12 months	Longitudinal cohort	Primary care centres	9.3, 4.4
Sweden	Agnafors et al. [79]	EPDS ≥ 10 (Translated, validated)	1,707	3 months	Cohort	Child welfare centres	12.0
	Kerstis et al. [80]	EPDS ≥ 10 (Translated, validated)	305 couples	3 months	Cohort	Child health centres	16.5
*11 study sites (Belgium, Germany, Italy, Poland and Spain)*	*Grote et al. [81]*	*EPDS ≥ 13* (English)	*929*	*2, 3, 6 months postnatal*	*Cohort study*	*11 study sites in five countries (specific study setting was not mentioned)*	*6–8 (Germany and Spain)* *13–16 (Belgium, Poland and Italy)*
Australia and New Zealand							
Australia	Mcmahon et al. [82]	M.I.N.I (English)	434	4 months	Prospective cohort	Assisted reproductive technology (art) clinics	8.3
	Woolhouse et al. [83]	EPDS ≥ 13 (English)	1,507	3, 6, 12 months	Cohort	Public hospital	16.1
	Wynter et al. [84]	EPDS ≥ 9 (English)	172 couples	6 months	Cross sectional	Local government areas	12.2
	Mcmahon et al. [85]	M.I.N.I (English)	541	4 months	Prospective cohort	Assisted reproductive technology clinics	7.9
	***Austin et al. [86]***	***EPDS score ≥ 13 CIDI (English)***	***235***	***2, 4, 6–8 months***	***Prospective***	***Obstetric hospital***	***24.4, 32.8***
	*Brooks et al. [87]*	*EPDS ≥* 13 (English)	*4,838*	*4, 8, 12, 16, 20, 24 weeks*	*Large cohort and prospective longitudinal design*	*Obstetric sites*	*6.0–9.0*
	*Bilszta et al. [88]*	*EPDS ≥* 13 (English)	*1,958 urban 908 rural*	*6th week postnatal*	*Cohort study*	*Perinatal health services*	*Urban: 6.6* *Rural: 8.5*
	*Milgrom et al. [89]*	*EPDS ≥* 13 (English)	*12,361*	*6th week postnatal*	*A large prospective cohort study*	*Maternity hospital antenatal clinics*	*7.5*
*New Zealand*	*Abbott and Williams [90]*	*EPDS ≥ 13* (Translated, validated by the researcher)	*1,376*	*6 weeks*	*Cross-sectional*	*Hospital and home visits*	*Samoans: 7.6* *Tongans: 30.9*
Asia							
Armenia	Petrosyan et al. [91]	EPDS ≥ 12 (Translated, validated by the researcher)	437	1–3 months	Case-control	Primary health care	14.4
*Bahrain*	***Al-Dallal et al. [92]***	***EPDS ≥ 12 (Translated, validated by the researcher)***	***237***	***8 weeks***	***Cross-sectional***	***Primary health care centres***	***37.1***
Bangladesh	Edhborg et al. [93]	EPDS ≥ 10 (Translated, validated)	672	2–3 months	Cohort	Community setting	11
	*Gausia et al. [94]*	*EPDS ≥ 10* (Translated, validated by the researcher)	*346*	*6–8 weeks*	*A community-based cohort study*	*Matlab subdistrict of rural Bangladesh*	*22*
China	Deng et al. [95]	EPDS ≥ 10 (Translated, validated)	1,823	4 weeks	Cross-sectional	Tangxia community	27.4
	Wu et al. [96]	EPDS ≥ 11 (Translated, validated)	223	3 months	Longitudinal	Obstetrics and gynaecology outpatient ward	9.4
	Mao et al. [97]	EPDS ≥ 13 (Translated, validated)	376	6–8 weeks	Cross-sectional	Postpartum clinics	14.9
Hong Kong	Ngai et al. [98]	GHQ ≥ 5 (Translated, validated)	200	6 months	Longitudinal	Regional hospital	11.5
	Lau et al. [99]	EPDS ≥ 10 EPDS ≥ 15 (Translated, validated)	610	6 weeks	Longitudinal	Obstetric outpatient clinics	31.6 8.7
India	Bodhare et al. [100]	PHQ-9 (Translated, validated)	274	6–8 weeks	Cross-sectional	Obstetrics and gynaecology clinic of a teaching hospital	39.8
	Johnson et al. [101]	EPDS ≥ 13 (Translated, validated by the researcher)	123	6–8 weeks	Cross-sectional	Maternity hospital	46.9
	Shivalli and Gururaj [102]	EPDS ≥ 13 (Translated, validated)	102	4–10 weeks	Cross-sectional	Obstetrics and gynaecology clinic	31.4
	Gupta et al. [103]	PRIME MD Today (PRIMary care evaluation of mental disorders) (Translated, validated)	202	6 weeks	Cross sectional	Postnatal clinic	15.8
Iran	Abdollahi et al. [104]	EPDS ≥ 13 (Translated, validated)	1,910	3 months	Longitudinal cohort	Primary health centres	19
	Hosseni et al. [105]	EPDS ≥ 13 (Translated, validated)	400	6 to 12 weeks	Cross-sectional	Health centres	40.4
	Abbasi et al. [106]	EPDS ≥ 13 (Translated, validated)	416	3 months	Prospective longitudinal	Teaching university hospitals	34.1
	Sadat et al. [107]	EPDS ≥ 13 (Translated, validated)	300	2 months and 4 months	Prospective	Health centres	22.3 15.7
	Goshtasebi et al. [108]	EPDS ≥ 13 (Translated, validated)	254	4–6 weeks	Prospective study	Hospital	5.5
	***Taherifard et al. [109]***	***EPDS ≥ 13 (Translated, validated)***	***179***	***6–8 weeks***	***Cross-sectional***	***Obstetrics and gynaecology clinics***	***34.8***
	Rouhi et al. [110]	EPDS ≥ 13 (Translated, validated)	436	8 weeks	Cross-sectional	Health care centres	36.3
	Kheirabadi et al. [111]	EPDS ≥ 13(Translated, validated)	1,898	6 to 8	Prospective cohort	Health centres	26.3
Israel	Alfayumi et al. [112]	EPDS ≥ 10 (Translated, validated)	564	4 weeks–7 months	Cross-sectional	Maternal and child health clinics	31
	Glasser et al. [113]	EPDS ≥ 10 (Translated, validated)	2,326	6 weeks	Prospective cohort	Maternal and child health clinics	16.3
Japan	Shimizu et al. [114]	EPDS ≥ 9 (Translated, validated)	65	1, 4 months	Prospective cohort	Obstetrics clinics	16.9, 7.7
	Matsumoto et al. [115]	EPDS ≥ 9 (Translated, validated)	675	≥ 3 months	Cohort	University hospital and maternity clinic	14.8
	***Miyake et al. [116]***	***EPDS ≥ 9 (Translated, validated)***	***771***	***3–4 months***	***Prospective cohort***	***Municipality of the domicile of the conception***	***13.8***
	Mori et al. [117]	EPDS ≥ 9 (Translated, validated)	675	5–7, 8–12 weeks	Cohort	University hospital	4
*Jordan*	***Mohammad et al. [118]***	***EPDS ≥ 13 (Translated, validated by the researcher)***	***353***	***6–8 weeks*** ***6 months***	***Prospective cross-sectional***	***Teaching hospital and health clinics***	***22.1, 21.2***
Korea	Park et al. [119]	EPDS ≥ 10 (Translated, validated)	153	4 weeks	Longitudinal	Maternity clinics	42.5
Lebanon	El-Hachem et al. [120]	EPDS ≥ 12 (Translated, validated)	149	30–40 postpartum	Cohort	Hospital	12.8
Malaysia	Yusuff et al. [121]	EPDS ≥ 12 (Translated, validated)	979	1, 3, 6 months	Prospective cohort	Maternal and child health clinics	14.3
	***Zainal et al. [122]***	***M.I.N.I (English)***	***411***	***6–8 weeks***	***Cross-sectional***	***Postnatal clinic, university hospital***	***6.8***
	*Kadir et al. [123]*	*EPDS ≥ 12* (Translated, validated)	*293*	*4–6 weeks*	*Cross sectional study*	*Postnatal clinic, hospital*	*27.3*
	*Azidah et al. [124]*	*EPDS ≥ 12* (Translated, validated)	*421*	*4–6 weeks*	*Cross sectional study*	*Maternal and child health clinics*	*20.7*
	*Wan Mohd Rushidi et al. [125]*	*EPDS ≥ 12 BDI-II ≥ 10 CIDI HDRS ICD-I0* (Translated, validated)	*354*	*4–12 weeks*	*A two-stage population survey*	*Health centres*	*16.38*
*Mongolia*	*Pollock et al. [126]*	*WHO Self Reporting Questionnaire (Translated, validated)*	*1,044*	*5 to 9 weeks postnatal*	*Cross-sectional*	*Hospital/home visit*	*9.1*
Nepal	Giri et al. [127]	EPDS ≥ 10 (Translated, validated)	346	6, 10 weeks	Cross-sectional	Maternity and women's hospital	30
	Budhathoki et al. [128]	EPDS ≥ 13 (Not mentioned)	72	6, 10 weeks	Prospective cohort study	Teaching hospital and district hospital	19.4, 22.2
	*Ho-Yen et al. [129]*	*EPDS ≥ 13* (Translated, validated)	*426*	*5–10 weeks postnatal*	*Cross-sectional structured interview study*	*Hospital's postnatal clinic, rural health posts, wards*	*4.9*
Oman	Al Hinai and Al Hinai [130]	EPDS ≥ 13 (Translated, validated)	282	8 weeks	Prospective cohort	Primary healthcare facilities	10.6
*Pakistan*	***Husain et al. [131]***	***EPDS ≥ 12 (Translated, validated)***	***763***	***≥ 3 months***	***Cohort study***	***Maternity and child care centre***	***38.3***
	*Muneer et al. [132]*	*EPDS ≥ 12* (Translated, validated)	*154*	*6 weeks postnatal*	*Cross sectional study*	*Outpatient sample*	*33.1*
*Qatar*	***Bener et al. [133]***	***EPDS ≥ 12 (Translated, validated)***	***1,379***	***6 months***	***Prospective cross-sectional study***	***Primary healthcare centres***	***17.6***
Saudi Arabia	Alasoom and Koura [134]	EPDS ≥ 10 (Translated, validated)	450	2–6 months	Cross-sectional	Primary healthcare centres	17.8
Taiwan	Tsao et al. [135]	EPDS ≥ 13 (Translated, validated)	162	6 weeks	Longitudinal cohort	Postnatal clinic at regional hospitals	24.1
	Lee et al. [136]	BDI-II ≥ 14 (Translated, validated)	60	5–8 weeks	Cross-sectional	Infertility treatment centre	25
Turkey	Bolak Boratav et al. [137]	EPDS ≥ 12 (Translated, validated)	87	3–6 months	Longitudinal	Obstetrics and gynaecology clinic	48.3
	Cankorur et al. [138]	EPDS ≥ 13 (Translated, validated)	578	2, 6 months	Cohort	Mother and child centres	26.1
	Kirkan et al. [139]	EPDS ≥ 13 (Translated, validated)	360	6 weeks	Prospective	City centre	13.3
	Turkcapar et al. [140]	EPDS ≥ 14 (Translated, validated)	540	6–8 weeks	Prospective	Specialized tertiary obstetrics and gynaecology hospital	15.4
	Annagur et al. [141]	EPDS ≥ 13 (Translated, validated)	197	6 weeks	Prospective	University hospital	14.2
	Poçan et al. [142]	EPDS ≥ 13 (Translated, validated)	187	4–6 weeks	Cross-sectional	University hospital	28.9
	***Kirpinar et al. [143]***	***EPDS ≥ 13 (Translated, validated)***	***479***	***6 weeks***	***Prospective***	***Primary heath care centres***	***14***
	Akyuz et al. [144]	PDSS ≥ 65 (Translated, validated)	156	4–6 weeks	Cohort	Hospitals	19.9
	*Dindar and Erdogan [145]*	*EPDS ≥ 12* (Translated, validated)	*679 mothers*	*1–12 months*	*Descriptive design*	Public health centres	*25.6*
UAE	Hamdan and Tamim [146]	EPDS ≥ 10, MINI (Translated, validated)	137	2 months	Prospective	Maternal and child health centre	5.9, 10.1
	*Green et al. [147]*	*EPDS ≥ 13* (Translated, validated)	*86, 56*	*3, 6 months*	*Longitudinal study*	Government maternity hospital	*22, 12.5*
Vietnam	Murray et al. [148]	EPDS ≥ 13 (Translated, validated)	431	1–6 months	Cross-sectional	Commune health centre	18.1

*Italic*: From initial review only.

**Bold**: Duplicates (both in initial and updated review).

## 4. Discussion and conclusion

This review found that the prevalence of PND ranged from 4.0–63.9% with Japan and America recording the lowest and highest rates of PND, respectively [9],[10]. Within continents, a wide variation in reported prevalence was also found. This finding is consistent with an earlier finding of a review of 143 studies across 40 countries that identified that the prevalence of PND ranged from 0–60% [21]. As with that review, this present review also indicated that the widely-cited prevalence of PND of 10–15% [149] does not represent the actual magnitude of PND problems worldwide. However, it should be noted that prevalence reported within this review was mainly based on self-report measures. Self-report measures have been found to give higher prevalence estimates than diagnostic tools [150]. This could explain the higher range of the prevalence reported in this study compared to the previous review.

Prevalence of PND can also vary depending on when the assessment is performed. For instance, assessing depressive symptoms in the postnatal period may inadvertently capture the common physiological or emotional responses to pregnancy and caring for an infant. Therefore, selective use of specific tools to screen women at higher risk for postpartum depression is recommended [151].

The willingness of a woman to admit to symptoms of PND may also influence the reported prevalence and this can vary across cultures as the label of PND may be unacceptable in some groups and may not be used at all [19]. Studies that have aimed to understand women's experiences and perceptions of PND have suggested that the majority of women were reluctant to disclose their depressive symptoms to healthcare providers [12],[13],[152]. There were many reasons why women did not reveal their inner turmoil but these were commonly linked with the stigmatization of a PND diagnosis, such as concern that it would make them “feel weak”, fear they would be judged as a “bad mother”, and fear of having their children referred to social services [152]. Stigma related to PND was found across a range of cultures and appeared to contribute to the women's feelings of being “viewed differently” from other mothers in their culture [153]–[155].

Although the reasons for the wide range of prevalence shown in this present review may also be explained by inconsistency in the estimated sensitivity and specificity of the EPDS (used in the majority of the studies in this review), it could also be linked to cross-cultural differences and the way in which women understand and interpret items in the EPDS. The EPDS was designed specifically by Cox et al. [156] to identify symptoms of PND. The EPDS consists of 10 statements describing depressive symptoms with some reverse coded items with four possible responses, 0, 1, 2, and 3, with each score relating to PND symptoms severity or duration. The total score is calculated by adding together the scores for each of the ten items with an overall score ranging from 0 to 30. Cut off scores for screening are typically set at ≥ 10 or ≥ 13. The reliability and validity of the Malay version of the EPDS has been verified [157],[158]. It was found to have good internal consistency with Cronbach's alpha 0.86, and split half reliability with Spearman split half coefficient 0.83 [158]. The score of 11.5 represents the optimum cut-off point for 72.7% sensitivity, 95% specificity, and a positive predictive value of 80% [157]. The cut off of 11/12 was recommended to identify a woman at risk of developing PND [121],[158].

Whilst it is understandable that the process of translation of the instruments and attempts to maintain the homogeneity of the interpretation of the questions had been considered, some cultures may define unique clusters of symptoms that differ from the western concept of PND [159]. Malaysia, a multi-ethnic country located in Southeast Asia comprises of three main races, including Malay (53.3%), Chinese (26.0%) and Indian (7.7%) [160]. Malaysia has a wide-range of cultural and ethnic backgrounds and this offers an ideal opportunity to understand the different role of cultures and postnatal practices in relation to PND. There are some small differences in postnatal practices among the three main cultures in Malaysia, such as in defining the period of the confinement. Within the Malaysian communities, the postnatal period is commonly referred to as postnatal confinement. In Malay society, the postnatal period is called *masa dalam pantang* [161] and both mother and baby are expected to remain house-bound for around 44 days. In Chinese communities, the postnatal period is the point from the baby's birth up to one month later, whereas the postpartum period in the Indian community refers to the period after the childbirth until between 30 and 40 days later [162]. Given that Malaysian's women have different cultural backgrounds compared to western cultures, the standard measurements that have been developed within western cultures like the EPDS, may not capture the localised expressions of depressive symptoms, and therefore lack conceptual equivalence. It may be possible that the women across cultures have different explanations of their PND experience which may go beyond the 10 items included in the EPDS.

Although there are questionnaires available to assess postnatal mental illness, these were generated based on western women's experiences which may not fully represent the signs and symptoms experienced by Malaysian women. Using any of these tools to detect postnatal mental illness among Malaysian women may therefore not be valid.

The prevalence of PND in Malaysia ranged from 6.8–27.3%, which has shown that the cases of PND were not as low as had initially been reported by two earlier reviews [20],[21]. In their international review of prevalence of PND, Halbreich and Karkun [21] reported that there were very few reports of PND in Malaysia with a rate of only 3.9%. Similarly, Klainin and Athur [20] stated that the prevalence of PND in Malaysia was only 3.5%, the lowest prevalence reported in Asian countries. Their finding was based on the review of 64 studies from 17 Asian countries conducted between 1998 and 2008. Both reviews presented their findings based on the only one Malaysian study published in 1997 [163]. This study was the earliest published study on the prevalence of PND in Malaysia. Kit et al. [163] conducted their study among 154 postnatal women from three main Malaysian cultural backgrounds; Malay, Chinese and Indian and reported that the rate of PND in Malaysia was 3.9%. There was a recent review by Norhayati et al. [164] that reported that the prevalence of PND in Malaysia at 4–6 weeks was 20.7%, but they also based this on only one study. The prevalence of PND reported in this present review was based on the results of five current studies in Malaysia, and this may increase confidence in the findings.

It seems clear that the rates of PND in Malaysia are not as low as reported by the international and Asian studies. Yet it is still unclear whether the wide range of reported prevalence of PND is due to variation in actual cases or to incorrect reports caused by use of instruments to diagnose PND that are inappropriate to the population and culture in Malaysia where, for example, there may be the stigma of a socially unacceptable reaction. Although the instruments used in the studies of the prevalence of PND (such as the EPDS) were translated into the Malay language items covered in these instruments may not fully match Malaysian understanding of PND. Therefore, there is a need for a screening scale that can measure the symptoms of PND as experienced by Malaysian women.

Despite contributing to understanding of the scale of PND problems across 50 countries, this review has four limitations that should be addressed. First, it only included the English/Malay articles in the chosen databases, which may have limited the generalisability of the findings. Second, the methodological quality of the included papers was not assessed, therefore the time of assessment of PND and inclusion criteria (such as maternal age, presence of medical and obstetrical problems, and socioeconomic status) varied across the studies. However, an effort has been made to include only rates reported after four weeks postnatal, therefore minimising the possibility of the inclusion of postnatal blues instead of PND. Third, this study did not conduct a meta-analysis of the prevalence. Fourth, this study uses two different sets of search terms, which may have resulted in some missed studies during the much more limited original review.

Overall, the reported rates of PND in Malaysia are much higher than that previously documented with a range of 6.8–27.3%. The reasons of this variability may not be fully explained using review methods. It is unclear whether variation is due to variation in actual cases or to the use of inappropriate instruments in assessing PND. This review recommends a meta-analysis study and a complementary qualitative study that could explain the nature of PND experience in Malaysia and address reasons for reported variation in prevalence.
